# Mental health status of adults under institutional quarantine: a cross-sectional survey in Tunisia

**DOI:** 10.11604/pamj.2021.40.197.31112

**Published:** 2021-12-01

**Authors:** Ghassen Kharroubi, Ines Cherif, Sina Haj Amor, Mariem Zribi, Wejdene Ben Atigue, Uta Ouali, Jihene Bettaieb

**Affiliations:** 1 Laboratory of Medical Epidemiology, Pasteur Institute of Tunis, Tunis, Tunisia,; 2 Laboratory of Transmission, Control and Immunobiology of Infections (LR11IPT02), Pasteur Institute of Tunis, Tunis, Tunisia,; 3 Faculty of Medicine of Tunis, University of Tunis El Manar, Tunis, Tunisia,; 4 Carthage Health District, Tunis, Tunisia,; 5 Department Psychiatry A, Razi Hospital, La Manouba, Tunisia

**Keywords:** Mental health, quarantine, depression, anxiety, insomnia, COVID-19

## Abstract

**Introduction:**

restrictive measures imposed during the COVID-19 pandemic, such as quarantine, may induce psychiatric outcomes among concerned individuals. The present study aimed to describe the mental health status of Tunisian adults under mandatory institutional quarantine imposed during the COVID-19 pandemic, and to determine factors influencing the occurrence of psychiatric symptoms.

**Methods:**

a cross-sectional phone survey was conducted from April to November 2020 using convenience sampling of persons who had experienced mandatory institutional quarantine. A standardized questionnaire was administered to participants including questions about socio-demographic characteristics and quarantine related information. Generalized anxiety disorder, depression symptoms, and sleep quality during quarantine were assessed using, respectively, the generalized anxiety disorder-7 (GAD-7), the centre for epidemiological studies depression (CES-D-10) and the insomnia severity index (ISI) scales. Bivariate and multivariate analyses were performed to determine factors associated with anxiety and/or depression and with clinical insomnia.

**Results:**

among 506 participants, 38.3% experienced anxiety and/or depression symptoms (anxiety: 15.4%; depression: 37.4%) and 19.2% had clinical insomnia. The prevalence of anxiety and/or depression symptoms and insomnia were higher among women those who spent three hours or above on COVID-19 news, those who had economic difficulties due to COVID-19 pandemic, those who were not satisfied by the accommodation conditions of quarantine facilities, and those who had experienced stigma.

**Conclusion:**

high prevalence of psychiatric symptoms among quarantined individuals was found in this study. Psychological interventions should thus be an integral part of the COVID-19 control strategy in order to provide adequate psychological support to persons quarantined due to COVID-19.

## Introduction

Shortly after its outbreak, the coronavirus disease (COVID-19) spread into a global pandemic. Since no effective medications or vaccination were available to combat the new virus at the early stage of the epidemic, many countries (at least 186) implemented varying degrees of restrictions on population movement to slow the spread of the SARS-CoV-2 and prevent healthcare facilities from becoming overwhelmed [1]. Most of these restrictive measures were unfamiliar for the general population, and even terms such as quarantine and isolation were often used interchangeably [2,3]. It is important to distinguish between them. Indeed, quarantine is defined as the separation and restriction of movement of people who have potentially been exposed to a contagious disease to ascertain if they become unwell and to reduce their risk of transmitting the virus. However, isolation is the separation of people who have been diagnosed with a contagious disease from people who are not sick [3,4].

In order to limit the spread of the SARS-CoV-2, Tunisian health authorities imposed a 14-day quarantine on travelers, close contacts of COVID-19 confirmed cases as well as on individuals coming from epidemic areas in Tunisia. While persons having contact with confirmed cases have to undergo a 14-day mandatory quarantine in their home, institutional quarantine was imposed for a short period during the first wave of the pandemic on individuals returning from epidemic areas in Tunisia. For travelers, terms and procedures of quarantine varied over time. Depending on epidemiological situation in foreign countries, Tunisian health authorities imposed either a 14-day quarantine in dedicated centers, 7-days of institutional quarantine followed by 7 days at home or a 14-day quarantine at home. Imposition of restrictive public health measures such as quarantine that infringe on personal freedoms and may induce financial losses is considered as a major stressor that contributes to widespread emotional distress and increased risk for psychiatric illness [5]. Indeed a systematic review found that those who experienced quarantine for at least one week were at higher risk for developing adverse mental health outcomes [6]. In addition institutional mandatory quarantine may be a source of additional stress due to environmental change. Conducting surveys to determine the frequency of psychiatric outcomes among quarantined persons and their predictors is necessary and useful to implement appropriate psychological interventions. In this context, our study aimed to describe the mental health status of Tunisian adults under mandatory institutional quarantine imposed during the COVID-19 pandemic and to determine factors influencing the occurrence of psychiatric symptoms.

## Methods

**Study design:** a cross-sectional phone survey was conducted from April 14^th^ to November 26^th^, 2020 among individuals under institutional quarantine.

**Study population and sample:** we used convenience sampling of persons who were placed in compulsory institutional quarantine in Tunisia. Individuals were eligible if they were 18 years or older, were in quarantine for 14 days, including at least 7 days in an approved quarantine center, and were consenting to participate in the study. Lists of persons complying with the inclusion criteria and their phone numbers were provided by the regional health directorates responsible for supervising the quarantine centers. Included individuals were contacted by phone each week from Monday to Friday during working hours. To optimize contact with persons unreachable at first call, at least one other attempt was made to contact them on a different day and at a different time of the day. On each call, investigators explained the aim of the study and that data will be treated anonymously. Those who gave their oral consent to participate, were interviewed.

**Data collection:** a standardized questionnaire was administered to participants including questions about socio-demographic characteristics and quarantine related information, such as stigma experience during the quarantine period explained by negative attitudes and believes regarding quarantined people leading to labelling and discrimination [7]. Participants were also asked about whether they had financial difficulties due to the COVID-19 pandemic (job loss, reduction in working hours, pay cut) and about their satisfaction by the accommodation conditions (food quality, hygiene, available furniture...) in quarantine centers. Generalized anxiety disorder, depression symptoms, and sleep quality were assessed using the three following scales: 1) the generalized anxiety disorder - 7 (GAD-7) scale which includes 7 items assessing the frequency of anxiety during the two weeks of quarantine according to a Likert scale (0: not at all; 1: several days; 2: more than half of the days; 3: nearly every day). The overall score varies from 0 to 21 points. A cut-off of 10 points indicates the presence of significant symptoms of anxiety [8]. The 10-item CES-D (center for epidemiologic studies depression) scale, which is made up of 10 items indicating the frequency of depression symptoms over a week during the quarantine period according to a Likert scale (rarely or none of the time/some or a little of the time / occasionally or a moderate amount of time/ most or all of the time). For each item, a score is assigned ranging from 0 to 3. The overall score varies from 0 to 30 points; a score of 10 points or more indicates the presence of significant depression symptoms [9]. The insomnia severity index (ISI) which includes 7 items assessing the nature, severity and impact of insomnia during the two weeks of quarantine. For each item, a score is assigned ranging from 0 to 4. This overall score varies from 0 to 28. A cut-off of 15 points indicates the presence of clinical insomnia (moderate to severe), while a score from 0 to 7 indicates the absence of insomnia, and a score from 8 to 14 reflects a sub-clinical insomnia [10].

**Data analysis:** data analysis was performed using Epi Info TM 7. Categorical variables were summarized in terms of counts and percentages. Prevalence of anxiety, depression symptoms and clinical insomnia were calculated considering the aforementioned cut-offs. In order to determine factors associated with anxiety or depression and with clinical insomnia, the chi square test in bivariate analysis and a backward stepwise multivariable logistic regression in multivariate analysis were used. Variables with a p-value equal or under 0.25 in bivariate analysis were included in the initial multivariable model. A p-value ≤0.05 was considered as statistically significant.

**Ethical considerations:** this study was conducted in accordance with the declaration of Helsinki, and was approved by the Biomedical Ethics Committee of the Pasteur Institute of Tunis (reference: 2020/21/I/LR16IPT).

## Results

**Participants´ socio-demographic characteristics, past medical history and quarantine related information:** in this study, 1340 eligible individuals were identified of whom 799 could not be reached after two calls and 35 refused to be interviewed. Finally, 506 persons accepted to participate in our study. The average period between the completion of the quarantine period and the interview was equal to 13.9±7.3 weeks. Respondents´ characteristics are presented in Table 1. Majority of participants (96.2%) were travelers and 83.4% spent the entire 14-day quarantine period in approved quarantine centers. Nearly two third of participants were men, their age ranged from 18 to 83 years and the mean age was equal to 37.6±13.6. Almost 57% had higher education, 59.1% were employees and 22.7% were students. Only 6.5% had a psychiatric history, among them more than half (54.5%) were taking long term psychiatric treatment.

**Table 1:** distribution of study respondents by socio-demographic characteristics, past medical history and quarantine related information (n=506)

Socio-demographic characteristics	n (%)
**Gender**	
Women	170 (33.6)
Men	336 (66.4)
**Age class (years)**	
18-39	302 (59.7)
40-59	166 (32.8)
≥60	38 (7.5)
**Educational level**	
Less than high school	75 (14.8)
High school or professional training	142 (28.1)
University	289 (57.1)
**Occupational status**	
Student	115 (22.7)
Employee	299 (59.1)
Unemployed/retired	92 (18.2)
**Marital status**	
Single	226 (44.7)
Married	258 (51.0)
Divorced / widowed	22 (4.3)
**Used to living alone**	
Yes	178 (35.2)
No	328 (64.8)
*Past medical history	
**Medical history of chronic disease**	
Yes	71 (14.0)
No	435 (86.0)
**Psychiatric history**	
Yes	33 (6.5)
No	473 (93.5)
*Quarantine related information	
**Quarantine modality**	
Fourteen days in a quarantine facility	422 (83.4)
Seven days in a quarantine facility and 7 days at home	84 (16.6)
**Quarantine reason**	
Arrival from a foreign country	487 (96.2)
Return from an epidemic area in Tunisia	19 (3.8)
**Access to media during quarantine**	
Yes	385 (76.1)
No	121 (23.9)
**Access to communication means (phone, internet) during quarantine**	
Yes	476 (94.1)
No	30 (5.9)
**Time spent following COVID-19 related news per day during quarantine**	
Less than 1 hour	337 (66.6)
1 to 3 hours	115 (22.7)
More than 3 hours	54 (10.7)
**Fear of being infected in the quarantine facility**	
Yes	296 (58.5)
No	210 (41.5)
**Having experienced stigma during quarantine**	
Yes	97 (19.2)
No	409 (80.8)
**Having financial difficulties due to COVID-19 pandemic**	
Yes	157 (31.0)
No	349 (69.0)
**Being convinced that quarantine is effective**	
Yes	457 (90.3)
No	49 (9.7)
**Satisfaction by the accommodation conditions of the quarantine facility**	
Yes	382 (75.5)
No	124 (24.5)
**Having benefited from psychological support during quarantine**	
Yes	28 (5.5)
No	478 (94.5)

* included employees. unemployed and retired persons

**Prevalence of psychiatric symptoms:** the prevalence of anxiety and/or depression symptoms was 38.3% (37.4% of participants had depression symptoms and 15.4% had anxiety symptoms), and the prevalence of clinical insomnia was 19.2%.

**Factors associated with psychiatric symptoms:** factors associated with anxiety and/or depression symptoms and clinical insomnia in bivariate analysis are detailed in Table 2. The multivariable logistic regression analysis (Table 3) showed that being a woman, spending three hours or above on COVID-19 news, having experienced stigma, having financial difficulties due to the COVID-19 pandemic, and being unsatisfied by the accommodation conditions of the quarantine facilities were significantly associated with anxiety and/or depression symptoms and to clinical insomnia. In addition, the occurrence of anxiety and/or depression symptoms was significantly higher among young adults, students and those who were afraid of being infected in the quarantine facilities. Clinical insomnia was significantly more frequent among participants who spent the entire quarantine period in containment facilities.

**Table 2:** psychiatric symptoms by participants’ characteristics and quarantine related information, results of bivariate analysis

	Anxiety and/ or depression			Insomnia		
	n (%)	Crude OR (95% CI)	P-value	n (%)	Crude OR (95% CI)	P-value
**Quarantine modality**			0.006			0.002
Fourteen days in a quarantine facility	173 (41.0)	2.08 (1.23-3.54)		91 (21.6)	3.57 (1.51-8.46)	
Seven days in a quarantine facility and 7 days at home	21 (25.0)	1		6 (7.1)	1	
**Gender**			<0.001			0.001
Women	86 (50.6)	2.16 (1.48-3.15)		47 (27.6)	2.18 (1.39-3.43)	
Men	108 (32.1)	1		50 (14.9)	1	
**Age class (years)**			<0.001			0.184
18-39	134 (44.4)	6.78 (2.35-19.58)	<0.001	65 (21.5)	2.33 (0.79-6.81)	0.122
40-59	56 (33.7)	4.33 (1.46-12.80)	0.008	28 (16.9)	1.72 (0.56-5.25)	0.337
≥60	4 (10.5)	1		4 (10.5)	1	
**Educational level**			0.013			0.047
University	125 (43.3)	2.24 (1.27-3.97)	0.005	66 (22.8)	2.17 (1.03-4.59)	0.042
High school or occupational certificate	50 (35.2)	1.60 (0.86-2.99)	0.139	22 (15.5)	1.34 (0.58-3.09)	0.485
Less than high school	19 (25.3)	1		9 (12.0)	1	
**Occupational status**			<0.001			0.032
Student	64 (55.7)	2.52 (1.65-3.850)		30 (26.1)	1.71(1.04-2.79)	
Other*	130 (33.2)	1		67 (17.1)	1	
**Used to living alone**			0.232			0.092
No	132 (40.2)	1.26 (0.86-1.84)		70 (21.3)	1.52 (0.93-2.47)	
Yes	62 (34.8)	1		27 (15.2)	1	
**Psychiatric history**			0.048			0.033
Yes	18 (54.5)	2.02 (1.01-4.12)		11 (33.3)	2.25 (1.05-4.81)	
No	176 (37.2)	1		86 (18.2)	1	
**Time spent following COVID-19 related news per day during quarantine**			0.033			0.001
More than 3 hours	28 (51.9)	2.02 91.13-3.61)	0.017	20 (37.0)	3.15 (1.68-5.890	<0.001
1 to 3 hours	49 (42.6)	1.39 (0.91-2.15)	0.130	24 (20.9)	1.41 90.82-2.42)	0.207
Less than 1 hour	117 (34.7)	1		53 (15.7)	1	
**Fear of being infected in the quarantine facility**			<0.001			0.005
Yes	141 (47.6)	2.69 (1.83-3.96)		69 (23.3)	1.97 [1.22-3.19]	
No	53 (25.2)	1		28 (13.3)	1	
**Having experienced stigma during quarantine**			<0.001			<0.001
Yes	53 (54.6)	2.29 (1.46-3.58)		36 (37.1)	3.37 (2.05-5.51)	
No	141 (34.5)	1		61 (14.9)	1	
**Having financial difficulties due to COVID-19 pandemic**			0.053			0.150
Yes	70 (44.6)	1.46 (0.99-2.14)		36 (22.9)	1.40 (0.88-2.23)	
No	124 (35.5)	1		61 (17.5)	1	
**Being convinced that quarantine is effective**			0.494			0.079
No	21 (42.9)	1.23 (0.67-2.23)		14 (28.6)	1.80 (0.93-3.50)	
Yes	173 (37.9)	1		83 (18.2)	1	
**Satisfaction by the accommodation conditions of the quarantine facility**			<0.001			<0.001
No	71 (57.3)	2.82 (1.86-4.27)		41 (33.1)	2.87 (1.79-4.59)	
Yes	123 (32.2)	1		56 (19.2)	1	

* included employees; unemployed and retired persons

**Table 3:** results of logistic regression analysis of associated factors for anxiety and/or depression symptoms and clinical insomnia

	Anxiety and/or depression		Insomnia	
	Adjusted OR (95% CI)	P-value	Adjusted OR (95% CI)	P-value
**Quarantine modality**		na		0.004
Fourteen days in a quarantine facility	Na		4.09 (1.55-10.82)	
Seven days in a quarantine facility and Seven days at home	Na		1	
**Gender**		0.001		0.001
Women	2.21 (1.41-3.47)		2.44 (1.44-4.13)	
Men	1		1	
**Age class (years)**		0.008		na
18-39	5.83 (1.88-18.03)	0.002	na	
40-59	4.50(1.45-13.990	0.009	na	
≥60	1		na	
**Occupational status**		0.002		na
Student	2.46(1.38-4.37)		na	
Other*	1		na	
**Time spent following COVID-19 related news per day during quarantine**		0.004		0.004
More than 3 hours	2.89 (1.49-5.60)	0.002	3.29 (1.63-6.64)	0.001
1 to 3 hours	1.53 (0.93-2.51)	0.092	1.28 (0.70-2.34)	0.415
Less than 1 hour	1		1	
**Fear of being infected in the quarantine facility**		<0.001		na
Yes	2.49 (1.63-3.79)		na	
No	1		na	
**Having experienced stigma during quarantine**		0.001		<0.001
Yes	2.31 (1.38-3.87)		3.17 (1.82-5.53)	
No	1		1	
**Having financial difficulties due to COVID-19 pandemic**		<0.001		0.024
Yes	2.34 (1.46-3.74)		1.91 (1.09-3.34)	
No	1		1	
**Satisfaction by the accommodation conditions of the quarantine facility**		0.003		0.002
No	2.04 (1.28-3.25)		2.33 (1.37-3.980)	
Yes	1		1	

* included employees, unemployed and retired persons NA: not applicable

## Discussion

The present study aimed to describe the mental health status of quarantined individuals in dedicated centers during the COVID-19 pandemic and the factors influencing it. Among respondents, more than one third (38.3%) experienced anxiety and/or depression symptoms (anxiety: 15.4%; depression: 37.4%), and nearly one fifth (19.2%) had clinical insomnia. The prevalence of anxiety and/or depression symptoms and insomnia were higher among women, those who spent three hours or above on COVID-19 news, those who had economic difficulties due to the COVID-19 pandemic, those who were not satisfied by the accommodation conditions of quarantine facilities, and those who had experienced stigma. Many studies assessing mental health outcomes have been conducted during the COVID-19 pandemic in several countries [11-21], in which a wide range of depression and anxiety frequencies were reported: the prevalence of anxiety symptoms ranged between 15% [11] and 84% [21] and of depression symptoms between 6.2% [20] and 55.2% [21]. However, the prevalence of insomnia in our study was similar to those reported by Gualano *et al*. [16] and Lin *et al*. [19]. Such comparisons should be interpreted with caution because of differences in the study populations. Indeed, in the present work, mental health outcomes were assessed among individuals who stayed in quarantine facilities for at least one week, whereas other studies were mainly conducted among the general population during lockdown periods. Also, different self-reported measures were used to assess psychiatric symptoms and for the same tool, different cutoffs were used.

The high prevalence of people experiencing clinically significant symptoms of depression, anxiety and insomnia in the present study emphasizes the importance of mental health support services during the quarantine period, mainly in quarantine facilities. In fact, only 5.5% of surveyed individuals reported that they benefited from psychological assistance in quarantine sites. Quarantined individuals should not be left to themselves without any support, they should be regularly supervised and be given practical advice on how to overcome boredom and cope with stress and anxiety [3]. In Tunisia, a centralized psychological support system, which integrates psychiatrists, medical students, psychologists and social services, was launched in march 2020 [22]. A helpline service free of charge and accessible from all over the country was implemented to give social and psychological support to the general population. However, the majority of participants came from abroad so they were probably not aware of the availability of such services in Tunisia. The Tunisian psychological support unit should thus coordinate with the managers of quarantine facilities to provide tailored psychological support to those persons. In our study, depression symptoms were more prevalent than anxiety ones. This result is consistent with some other studies [12,15,16,19]. Given that depression might occur as a result of anxiety disorders [23] and most of participants came from abroad where they had been quarantined for several weeks during lockdown periods, they might thus have already experienced anxiety symptoms before they quarantined in Tunisia, which increases the risk for secondary depression [23]. Indeed, the GAD-questionnaire primarily screens for symptoms of generalized anxiety disorder, meaning that other anxiety symptoms might have been overlooked. In addition, in the Tunisian population, anxiety is more often expressed through somatic symptoms, such as feelings of thoracic oppression or suffocation, aches and pains, which is not evaluated by the GAD questionnaire [24].

After multivariate logistic regression analysis, psychiatric distress was more frequent among women. This gender difference was found in several other studies [11,13,14,16,19,25]. Indeed, women are known for being more likely to develop mood and anxiety disorders [26,27]. Moreover, when facing adverse events, women are more likely to expect danger and harm compared to men, and institutional quarantine could be considered as such (an adverse event) [28]. Furthermore, women have a higher tendency to ruminate on negative emotions [29]. In addition, we found that depression and/or anxiety symptoms were more frequent among younger individuals. This result is in agreement with those of other studies [11-14,17,18]. This may be explained by the fact that younger people are generally physically more active than older ones. Deprivation of liberty due to compulsory quarantine may therefore have a greater impact on them. Students were also more at risk of developing the aforementioned symptoms. Indeed, exam delays in universities, the new method of learning imposed by the COVID-19 pandemic and unpredictable future may be a source of frustration [14]. After controlling for other factors, psychiatric symptoms were more frequent among participants who spent three hours or above on COVID-19 news. Those results are in line with the findings of Huang Y *et al*. [18]. In fact, the World Health Organization (WHO) has recommended reducing time spent on news, which can be a source of anxiety and fear, and to rather seek information at most twice a day from reliable sources such as the WHO website or the platforms of healthcare authorities [30]. Instead, people in quarantine may fill their spare time with physical activities, which can decrease the negative psychological impact of quarantine [31]. In the present study, almost one fifth of participants have experienced stigma and those individuals were more likely to present anxiety and/or depression symptoms and clinical insomnia.

Training sessions for the staff working at quarantine facilities should thus be organized in order to provide them with updated information and ensure good quality communication between the staff and quarantined individuals [30]. We also found that experiencing economical struggle was independently associated with the occurrence of psychological symptoms, in line with the results of other studies [11,15,16]. Similarly, being dissatisfied by the accommodation conditions of quarantine facilities was associated with a higher risk of developing such symptoms. Having basic furniture in the rooms and acceptable food quality are indeed essential. The prevalence of anxiety and/or depression symptoms did not differ depending on whether the quarantine period was spent in whole or in part in a quarantine center. It is therefore important to provide psychological support to people as soon as they are admitted in quarantine facilities, targeting in priority the most vulnerable persons. However, the occurrence of clinical insomnia appeared to be more frequent in persons who spent the entire quarantine period in quarantine facilities. Home quarantine may have a lower impact on individuals´ mental health, but it is not possible to guarantee compliance with this quarantine modality in our country as it is difficult to implement effective measures to ensure that people are staying at home such as advanced tracking technologies. Institutional quarantine would be more appropriate also to limit the spread of new variants of SARS-CoV-2. Thus, Tunisian health authorities reimposed this procedure for all travelers since February 1^st^, 2021, shortly after the emergence of the new variants B.1.1.7 and B.1.351.

**Limitations:** first, the questionnaires used in this study for assessing general anxiety disorder, depression and insomnia only allow identification of probable cases. Therefore, we do not have diagnostic data. Second, as we did not dispose of the exhaustive lists of individuals who were under institutional quarantine in Tunisia, we carried out a non-probabilistic sampling which may affect data representativeness. Third, in phone surveys, there is a selection bias. Indeed we could not reach persons who refused to give their phone number, gave a wrong number to the staff in quarantine facilities or did not respond to their phone after two call attempts. Also we couldn't be one hundred percent sure that the person answering was the one who was in quarantine. Fourth, there may be a recall bias as the interview was done for some participants several weeks after the quarantine period. Finally, cross sectional studies cannot indicate the chronology of events and no causal relationship could thus be established between the identified associated factors and psychological symptoms.

## Conclusion

High prevalence of psychiatric symptoms among quarantined individuals was found in this study. Psychological interventions should thus be an integral part of the COVID-19 control strategy in order to provide adequate psychological support to persons in need, including those in quarantine sites. The provisions of regular updated and comprehensible information, in addition to adequate accommodation conditions are also necessary to guarantee a successful quarantine with a minimum of negative effects.

**Funding:** this work was supported by the Tunisian Ministry of Higher Education and Scientific Research.

### What is known about this topic

- In order to slow the spread of the SARS-CoV-2, many countries have implemented varying degrees of restrictions on population movement;
- Restrictive public health measures, such as quarantine, may lead to an increased risk for psychiatric illness.

### What this study adds

- To the best of our knowledge, the present study is the first to report the mental health status of Tunisian adults under compulsory institutional quarantine;
- This study provides benchmark data to use by health authorities in order to improve the mental health support in quarantine sites.
